# Early tumor shrinkage and response assessment according to mRECIST predict overall survival in hepatocellular carcinoma patients under sorafenib

**DOI:** 10.1186/s40644-021-00439-x

**Published:** 2022-01-04

**Authors:** Osman Öcal, Regina Schinner, Kerstin Schütte, Enrico N. de Toni, Christian Loewe, Otto van Delden, Vincent Vandecaveye, Bernhard Gebauer, Christoph J. Zech, Christian Sengel, Irene Bargellini, Antonio Gasbarrini, Bruno Sangro, Maciej Pech, Peter Malfertheiner, Jens Ricke, Max Seidensticker, for the SORAMIC study group, E. Kettner, H. Amthauer, J. Cwikla, J. Walecki, H. Klümpen, E. Schott, F. Kolligs, O. Rosmorduc, Y. Menu, V. Leroy, J. Mayerle, C. Trumm, P. Bartenstein, M. Reiser, T. Berg, M. Moche, I. Bilbao, L. Gossner, P. Reimer, P. Popovic, B. Stabuc, P. Piasecki, Z. Podgajny, R. Sacco, M. Peck-Radosavljevic, J. Lammer, G. Maleux, C. Verslype, C. Rosenberg, D. Nitsche, P. Waldenberger, J. Vergniol, C. Cassinotto, S. Yalcin, B. Peynircioglu, C. Zavaglia, A. Rampoldi, A. Tran, P. Chevallier, R. Anty, C. Trautwein, C. Kuhl, L. Grazioli, T. Vogl, J. Trojan, C. Bartolozzi, R. Iezzi, J. P. Bronowicki, D. Palmer, J. Evans, R. Sharma, G. Weir, R. Hubner, B. Basu, P. Ross

**Affiliations:** 1 Department of Radiology, University Hospital, LMU Munich, Marchioninistrasse 15, 81377 Munich, Germany; 2 Department of Internal Medicine and Gastroenterology, Niels-Stensen-Kliniken Marienhospital, Osnabrück, Germany; 3 Department of Internal Medicine II, University Hospital, LMU Munich, Munich, Germany; 4 Section of Cardiovascular and Interventional Radiology, Department of Bioimaging and Image-Guided Therapy, Medical University of Vienna, Vienna, Austria; 5 Department of Radiology and Nuclear Medicine, Academic Medical Center, University of Amsterdam, Amsterdam, The Netherlands; 6 Department of Radiology, University Hospitals Leuven, Leuven, Belgium; 7 Department of Radiology, Charité – University Medicine Berlin, Berlin, Germany; 8 Radiology and Nuclear Medicine, University Hospital Basel, University of Basel, Basel, Switzerland; 9 Radiology Department, Grenoble University Hospital, La Tronche, France; 10 Department of Vascular and Interventional Radiology, University Hospital of Pisa, Pisa, Italy; 11 Gastroenterology, Gemelli Foundation, Catholic University, Rome, Italy; 12 Liver Unit, Clínica Universidad de Navarra, Pamplona, Spain; 13 Departments of Radiology and Nuclear Medicine, University of Magdeburg, Magdeburg, Germany

**Keywords:** Hepatocellular carcinoma, Sorafenib, mRECIST, Early tumor shrinkage, Objective response

## Abstract

**Background:**

The aim of this study was to explore the relationship between follow-up imaging characteristics and overall survival (OS) in advanced hepatocellular carcinoma (HCC) patients under sorafenib treatment.

**Methods:**

Associations between OS and objective response (OR) by mRECIST or early tumor shrinkage (ETS; ≥20% reduction in enhancing tumor diameter at the first follow-up imaging) were analyzed in HCC patients treated with sorafenib within a multicenter phase II trial (SORAMIC). 115 patients were included in this substudy. The relationship between survival and OR or ETS were explored. Landmark analyses were performed according to OR at fixed time points. Cox proportional hazards models with OR and ETS as a time-dependent covariate were used to compare survival with factors known to influence OS.

**Results:**

The OR rate was 29.5%. Responders had significantly better OS than non-responders (median 30.3 vs. 11.4 months; HR, 0.38 [95% CI, 0.22–0.63], *p* < 0.001), and longer progression-free survival (PFS; median 10.1 vs. 4.3 months, *p* = 0.015). Patients with ETS ≥ 20% had longer OS (median 22.1 vs. 11.4 months, *p* = 0.002) and PFS (median 8.0 vs. 4.3 months, *p* = 0.034) than patients with ETS < 20%. Besides OR and ETS, male gender, lower bilirubin and ALBI grade were associated with improved OS in univariate analysis. Separate models of multivariable analysis confirmed OR and ETS as independent predictors of OS.

**Conclusion:**

OR according to mRECIST and ETS in patients receiving sorafenib treatment are independent prognostic factors for OS. These parameters can be used for assessment of treatment benefit and optimal treatment sequencing in patients with advanced HCC.

**Supplementary Information:**

The online version contains supplementary material available at 10.1186/s40644-021-00439-x.

## Introduction

Hepatocellular carcinoma (HCC) is the most frequent primary liver cancer and the third leading cause of cancer-related death [1]. Approximately 60–80% of patients with newly diagnosed HCC have an underlying liver disease, including chronic hepatitis B or C infections, alcoholic liver cirrhosis, and non-alcoholic steatohepatitis. Despite screening populations at risk, only about 30% of patients are diagnosed at early stages that might benefit from potentially curative treatments.

Sorafenib is a multitarget tyrosine kinase inhibitor that interrupts tumor proliferation and angiogenesis, and was shown in the phase III SHARP trial and the Asia-Pacific trial to improve overall survival (OS) in HCC [2, 3]. Although OS and time to progression were improved in the sorafenib arm in both studies, the objective response (OR) rate according to RECIST was 2–3% and this failed to capture patients with survival benefit. To overcome this problem, mRECIST has been proposed for response assessment in patients with HCC, which, in contrast to RECIST, employs the arterially enhancing portion of the target lesions only [4]. Whereas some retrospective studies showed better OS in patients with OR according to mRECIST in patients receiving sorafenib, others failed to demonstrate improved outcomes [5–9]. One study which combined analysis of two prospective phase II studies showed patients with an OR had significantly longer survival; however, this significance was lost in multivariate analysis including macrovascular invasion and extrahepatic disease [10]. However, most of these studies were single-center and retrospectively conducted, and in none of them, statistical methods to exclude biased estimates of survival were applied [11]. Recently a subanalysis of the phase III SILIUS trial comparing sorafenib alone vs. sorafenib and hepatic arterial infusion chemotherapy in Japan demonstrated that OR was an independent prognostic factor for OS using appropriate statistical methods [12].

Although patients continue to receive their assigned treatment unless disease progression is encountered at follow-up imaging, not all patients with disease control benefit equally from treatment. A retrospective analysis of HCC patients receiving sorafenib revealed that patients who had stable disease (SD) for more than three months had similar OS to patients who had an OR, while patients with SD for a shorter duration had worse outcomes, similar to patients with progressive disease (PD) [6].

In clinical practice, early identification of patients benefitting from sorafenib treatment is crucial to avoid overtreatment, which may lead to toxicities or suboptimal treatment sequencing, especially in light of alternative treatments [13–15]. Early tumor shrinkage (ETS) is defined as a reduction in tumor size at the first radiological follow-up evaluation and it has been shown to predict treatment outcome in patients with metastatic colorectal carcinoma, pancreatic cancer, renal cell carcinoma, and also in patients with HCC receiving lenvatinib treatment [16–19].

This study aimed to evaluate the prognostic role of OR and ETS in patients receiving sorafenib therapy for the treatment of advanced HCC in a Western cohort.

## Material and methods

### Study population

SORAMIC was a prospective, randomized, controlled, phase II trial comparing the effects of sorafenib monotherapy and a combination of selective internal radiation therapy (Yttrium-90 radioembolization) and sorafenib, performed in 38 centers in 12 countries in Europe and Turkey. The inclusion criteria for SORAMIC have been described previously [20]. The main criteria were a diagnosis of HCC with Barcelona Clinic Liver Cancer (BCLC) B (not eligible for transarterial chemoembolization) or C, preserved liver function (Child-Pugh scores A to B7), and an Eastern Cooperative Oncology Group performance status ≤2. Extrahepatic metastases were permitted if the disease was liver-dominant and did not involve the lungs.

The present study represents a post hoc analysis of patients in the sorafenib-only arm of the palliative trial cohort. The study cohort comprised 208 patients randomized to receive sorafenib monotherapy. The study protocol was approved by the institutional review board of participating centers. Written, informed consent was obtained from all patients. The primary aim of the analysis was to explore the radiological response rate to sorafenib treatment and its correlation with OS. Criteria were OR, ETS, and depth of response (DpR).

Follow-up imaging every three months was recommended, but was not a mandatory part of the SORAMIC trial. The imaging modality – computed tomography (CT) or magnetic resonance imaging (MRI) – was chosen by the local investigator. Centralized image assessment was not included in the main study. Imaging follow-up of a total of 136 patients was available for review. The following inclusion criteria were applied for this study: (1) at least one follow-up before 6 months or 6 months follow-up other than PD, (2) follow-up imaging until death or PD (last imaging within 6 months) or disease control (SD, partial response [PR], complete response [CR]) at 12 months after randomization, (3) minimum follow-up duration of 6 months unless PD was encountered before. A total of 115 patients were included in this study (Supplementary Fig. 1).

### Sorafenib treatment

Patients were administered sorafenib with a starting dose of 200 mg b.i.d. for 1 week. After the first week, the dose was increased to the target dose of 400 mg b.i.d., and, in case of toxicity, the sorafenib dose was modified according to pre-defined dosing guidelines. The lowest accepted dose was 200 mg b.i.d. on alternate days. Following the resolution of toxicities, maintaining the highest tolerable dose level was attempted with a stepwise dose re-escalation. Treatment-related adverse events and routine laboratory tests were recorded every two months, and sorafenib treatment was continued until disease progression (evaluated by the local investigator) or toxicity which required discontinuation.

### Image analysis

All patients underwent CT and MRI at baseline for study inclusion according to previously published protocols [21]. Follow-up CT in 60 patients, MRI in 45 patients, and both MRI and CT in 10 patients were available. Review was performed by a fully blinded, board-certified radiologist specialized in gastrointestinal imaging. mRECIST was used for all assessments (Supplementary Fig. 2). Two liver lesions were selected as target lesions, according to published criteria [22]; in the case of extrahepatic disease at baseline, up to three extrahepatic lesions were selected as target lesions. As described in mRECIST, overall tumor diameter measurements were used in intrahepatic lesions with atypical enhancement patterns and all extrahepatic lesions.

In addition to routine response analysis according to mRECIST, progression-free survival (PFS; from randomization to disease progression or death, censored at last imaging in patients without progression), time to response (from randomization to first objective response), DpR (relation of smallest target lesion diameter to baseline diameter), ETS (≥20% reduction in enhancing tumor diameter at the first follow-up imaging), and time to DpR (from randomization to DpR) was evaluated. For DpR and ETS assessments, only hepatic target lesions were evaluated and diameter measurements were taken according to mRECIST. In patients with disease progression, the first progression site was noted (hepatic, extrahepatic, or both). In order to evaluate whether further subgrouping of patients translated to better survival prediction, patients with the best response of PR were dichotomized according to median DpR.

### Statistical analysis

All statistical analyses were performed using SAS version 9.4 for Windows (Copyright SAS Institute Inc., Cary, NC, USA) and R statistical and computing software, version 3.5.0 (http://www.r-project.org). Numerical data are presented as means with standard deviations. For categorical data, results are given as absolute numbers with percentages. For comparison of categorical data between responders and non-responders, chi-square tests were applied; for continuous data, T-tests or Mann-Whitney U tests were used for testing the homogeneity of independent samples. OS in responders and non-responders was estimated by the unweighted Kaplan-Meier method. The same analysis was repeated for patients with and without ETS. The Mantel-Byar test was used to assess statistical significance. Landmark analysis of OS by the objective response was conducted at 6 and 12 months after randomization. The log-rank test was used for the inference associated to the landmark analysis. For all variables, univariate Cox proportional hazard regression was performed as time-fixed covariates, while objective response and ETS were analyzed as a time-dependent variable. Statistically significant variables in the univariate analyses (including objective response as a time-dependent covariate) were analyzed in a multivariate Cox regression model to explore prognostic factors for OS. A separate multivariate Cox regression model was used to explore the prognostic value of ETS.

## Results

At the end of study, 93 (80.8%) patients had died, with a median OS of 14.3 months. Out of 115 patients, 34 (29.5%) were responders and the remaining 81 (70.4%) were non-responders according to mRECIST. Best response during follow-up was CR in 6 (5.2%) patients, PR in 28 (24.3%) patients, SD in 50 (43.4%) patients, and PD in 31 (26.9%) patients. Median time to response was 3.8 (range 1.3–8.1) months. The baseline and clinical characteristics of patients with and without OR are shown in Table 1. No significant differences were observed in the baseline characteristics of responders and non-responders.

**Table 1:** Patient characteristics of responders and non-responders

Variable	Total ***n*** = 115	Responders ***n*** = 34	Non-responders ***n*** = 81	***p***-value
**Sex**				
**Female**	15 (13.0%)	7 (20.6%)	8 (9.9%)	0.1196
**Male**	100 (87.0%)	27 (79.4%)	73 (90.1%)	
**Age, years**				
**Mean (SD)**	65.3 (8.7)	66.1 (7.8)	65.0 (9.1)	0.5156
**Median (IQR)**	65.0 (12.0)	67.0 (13.0)	65.0 (12.0)	
**Age category, years**				
**≤ 65**	55 (47.8%)	15 (44.1%)	40 (49.4%)	0.606
**> 65**	60 (52.2%)	19 (55.9%)	41 (50.6%)	
**Ethnicity**				
**Missing**	10 (8.7%)	4 (11.8%)	6 (7.4%)	0.6638
**Hispanic or Latin**	5 (4.8%)	1 (3.3%)	4 (5.3%)	
**Other**	100 (95.2%)	29 (96.7%)	71 (94.7%)	
**Race**				
**Missing**	11 (9.6%)	4 (11.8%)	7 (8.6%)	0.2326
**Black**	2 (1.9%)	0	2 (2.7%)	
**Other**	3 (2.9%)	2 (6.7%)	1 (1.4%)	
**White**	99 (95.2%)	28 (93.3%)	71 (95.9%)	
**ECOG status**				
**0**	90 (78.3%)	27 (79.4%)	63 (77.8%)	0.8463
**1**	25 (21.7%)	7 (20.6%)	18 (22.2%)	
**HCC diagnosis by:**				
**Missing**	2 (1.7%)	1 (2.9%)	1 (1.2%)	0.7518
**EASL criteria**	50 (43.5%)	13 (38.2%)	37 (45.7%)	
**Histology**	50 (43.5%)	15 (44.1%)	35 (43.2%)	
**Other**	13 (11.3%)	5 (14.7%)	8 (9.9%)	
**Hepatitis B**				
**No**	104 (90.4%)	31 (91.2%)	73 (90.1%)	0.8609
**Yes**	11 (9.6%)	3 (8.8%)	8 (9.9%)	
**Hepatitis C**				
**No**	81 (70.4%)	26 (76.5%)	55 (67.9%)	0.3581
**Yes**	34 (29.6%)	8 (23.5%)	26 (32.1%)	
**Alcohol etiology**				
**No**	63 (54.8%)	16 (47.1%)	47 (58.0%)	0.2809
**Yes**	52 (45.2%)	18 (52.9%)	34 (42.0%)	
**Previous therapies**				
**TACE**	28 (24.3%)	9 (26.5%)	19 (23.5%)	0.7311
**TAE**	2 (1.7%)	0	2 (2.5%)	0.3553
**Resection**	21 (18.3%)	9 (26.5%)	12 (14.8%)	0.1398
**RFA**	12 (10.4%)	6 (17.6%)	6 (7.4%)	0.1012
**Brachytherapy**	5 (4.3%)	1 (2.9%)	4 (4.9%)	0.6318
**Max. diameter of largest lesion**				
**Mean (SD)**	68.0 (59.6)	69.8 (85.5)	67.3 (44.6)	0.2678
**Median (IQR)**	57.0 (45.0)	50.0 (40.0)	59.5 (52.0)	
**Portal vein infiltration**				
**Yes**	60 (52.2%)	21 (61.8%)	39 (48.1%)	0.1822
**Baseline metastasis**				
**Yes**	6 (5.2%)	0	6 (7.4%)	0.1031
**BCLC**				
**B**	34 (29.6%)	9 (26.5%)	25 (30.9%)	0.6375
**C**	81 (70.4%)	25 (73.5%)	56 (69.1%)	
**Up-to-7 criterion**				
**Inside**	17 (14.8%)	7 (20.6%)	10 (12.3%)	0.2558
**Outside**	98 (85.2%)	27 (79.4%)	71 (87.7%)	
**Total bilirubin (μmol/L)**				
**Mean (SD)**	16.1 (7.1)	15.4 (7.3)	16.3 (7.1)	0.4752
**Median (IQR)**	14.9 (10.0)	14.4 (9)	15.0 (9.5)	
**Albumin g/L**				
**Mean (SD)**	37.8 (8.3)	38.9 (8.5)	37.4 (8.2)	0.3823
**Median (IQR)**	39.0 (7.7)	39.9 (6.5)	39.0 (8.0)	
**ALBI score**				
**Mean (SD)**	−2.5 (0.7)	−2.6 (0.8)	−2.4 (0.7)	0.2331
**Median (IQR)**	−2.5 (0.8)	−2.7 (0.7)	−2.5 (0.7)	
**Child-Pugh score**				
**A**	105 (91.3%)	33 (97.1%)	72 (88.9%)	0.1559
**B**	10 (8.7%)	1 (2.9%)	9 (11.1%	

*ALBI* albumin-bilirubin, *BCLC* Barcelona Clinic Liver Cancer, *EASL* European Association for the Study of the Liver, *ECOG* Eastern Cooperative Oncology Group, *HCC* hepatocellular carcinoma, *IQR* interquartile range, *RFA* radiofrequency ablation, *SD* standard deviation, *TACE* transarterial chemoembolization, *TAE* transarterial embolization

**Fig. 1:**
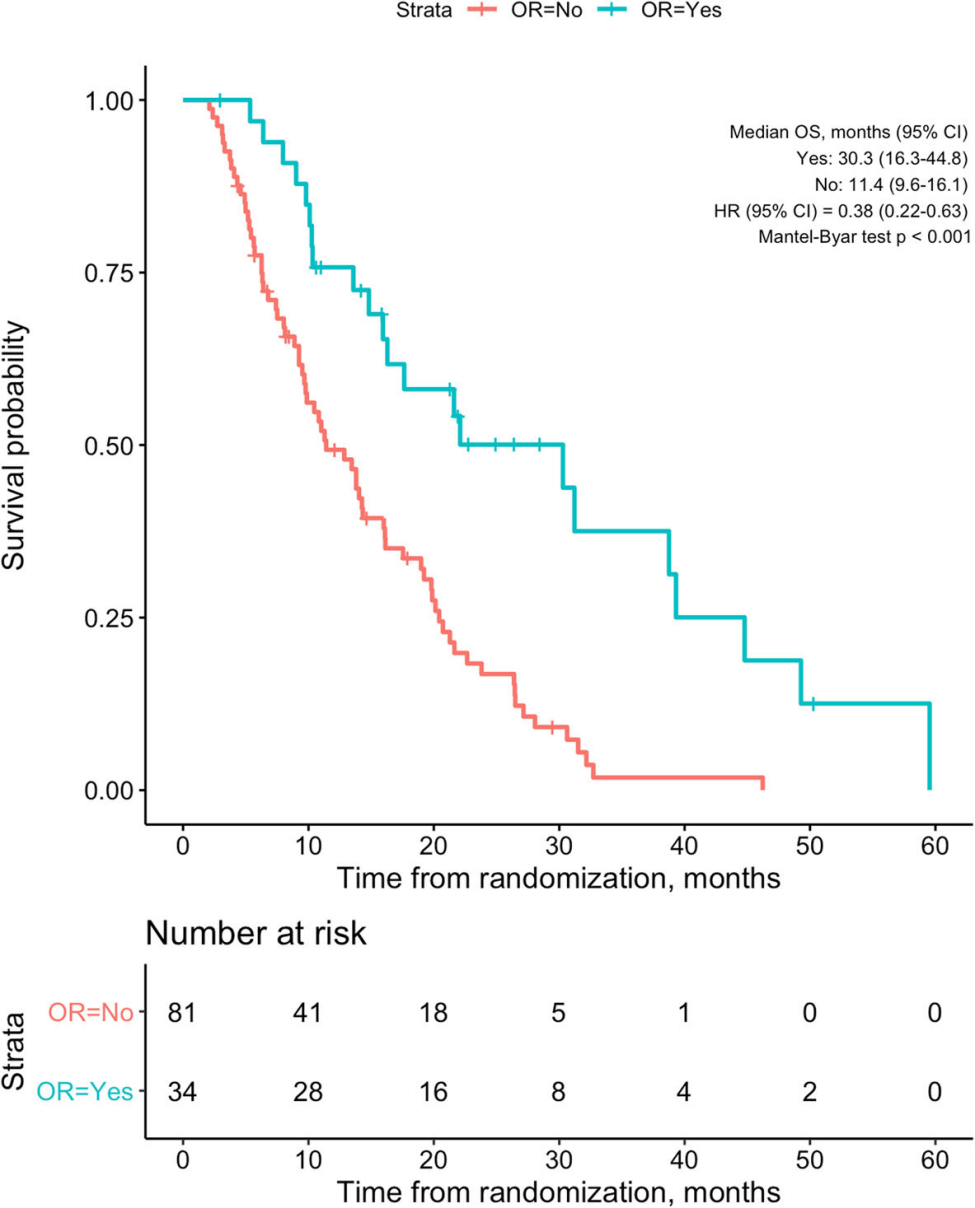
Overall survival of patients with, compared to patients without, objective response. CI, confidence interval; HR, hazard ratio; OR, objective response; OS, overall survival

The median OS was 30.3 months (95% CI, 16.3–44.8) in responders and 11.4 months (95% CI, 9.7–16.1) in non-responders (HR, 0.38 [95% CI, 0.22–0.63, *p* < 0.001) (Fig. 1). Landmark analyses at 6 and 12 months showed longer OS in responders compared to non-responders (Supplementary Figs. 3&4). According to best response by mRECIST, the median OS in patients with CR was 49.1 (95% CI, 38.8–59.5), PR 17.6 (95% CI, 14.8–44.8), SD 14.3 (95% CI, 12.9–20.4), and PD 8.0 (95% CI, 6.3–11.4) months (Supplementary Fig. 5). Patients with PR had significantly longer OS than patients with SD (HR, 0.56 [0.32–0.97], *p* = 0.037). Median DpR of patients with PR was 54.4% (range, 33–88.2). There was no significant OS difference between PR patients with a DpR greater than or lower than the median (HR, 1.09 [0.44–2.67], *p* = 0.854; Supplementary Fig. 6). Median time to DpR was 4.8 months in responders.

**Fig. 2:**
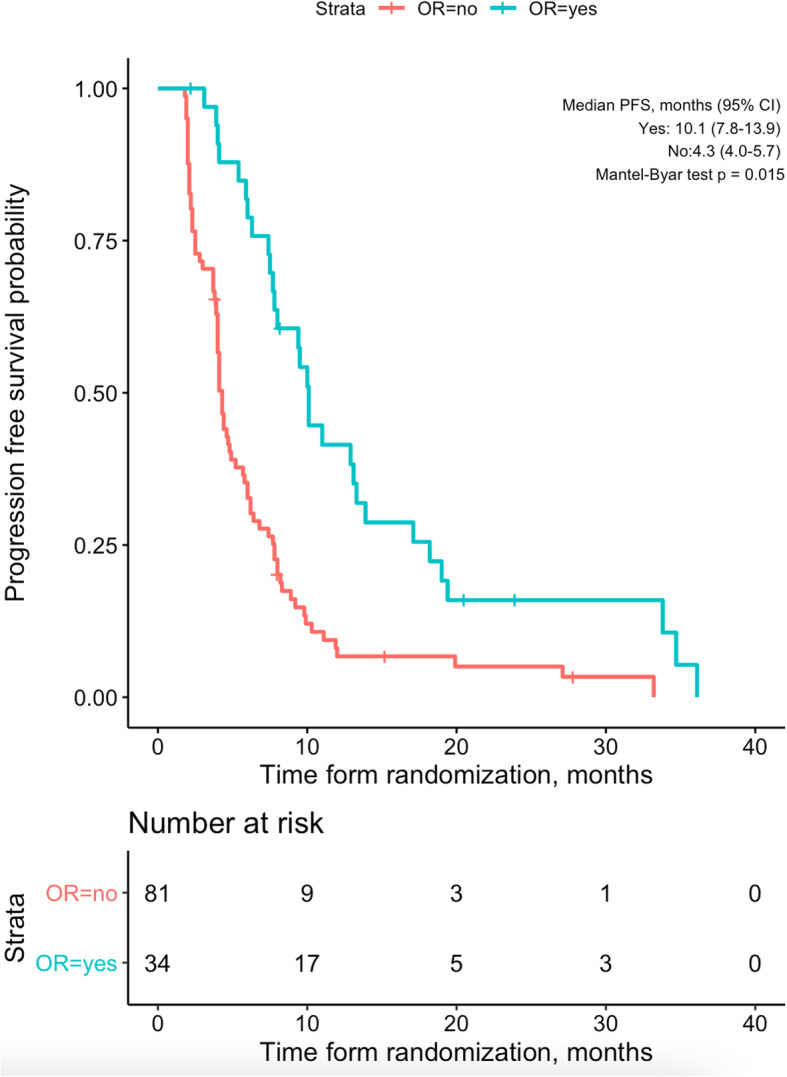
Progression-free survival of patients with, compared to patients without, objective response. CI, confidence interval; PFS, progression-free survival

Responders also had longer PFS than non-responders (10.1 vs. 4.3 months, *p* = 0.015; Fig. 2). Progression was encountered in 24 (70.6%) responders and 63 (77.8%) non-responders (*p* = 0.412). Although the difference is not significant, in none of the responders was the first progression extrahepatic (0/24 vs. 9/63, *p* = 0.058).

Besides OR, in univariate analysis, male gender (HR, 0.50 [95% CI, 0.27–0.91], *p* = 0.024) was associated with better OS, while bilirubin ≥17 μmol/L (HR, 1.55 [95% CI, 1.02–2.36], *p* = 0.039) and albumin-bilirubin (ALBI) grade 2–3 (HR, 1.74 [95% CI, 1.14–2.66], *p* = 0.009) were associated with poorer OS. Multivariate Cox regression analysis revealed that OR assessed by mRECIST was an independent prognostic factor (HR, 0.32 [95% CI, 0.18–0.55], *p* < 0.001). Male gender (HR, 0.41 [95% CI, 0.22–0.75], *p* = 0.004) and ALBI grade (HR, 1.88 [95% CI, 1.21–2.92], *p* = 0.005) were also independent prognostic factors for OS (Table 2).

**Table 2:** Univariable and multivariable analysis of factors associated with overall survival

Parameter	Univariate analysis		Multivariate analysis	
	HR (95% CI)	***p*** value	HR (95% CI)	***p*** value
**Best response (CR + PR vs. SD + PD)**	0.38 (0.22–0.63)	**< 0.0001**	0.32 (0.18–0.55)	**< 0.001**
**Sex (male vs. female)**	0.50 (0.27–0.91)	**0.0241**	0.41 (0.22–0.75)	**0.004**
**Age (≥65 vs. < 65 years)**	1.01 (0.67–1.54)	0.9310		
**ECOG (1 vs. 0)**	0.80 (0.48–1.35)	0.4184		
**Cirrhosis (yes vs. no)**	1.22 (0.63–2.37)	0.5457		
**Hepatitis B etiology (yes vs. no)**	1.22 (0.61–2.44)	0.5677		
**Hepatitis C etiology (yes vs. no)**	1.17 (0.74–1.85)	0.4847		
**Alcohol etiology (yes vs. no)**	0.78 (0.51–1.21)	0.2798		
**TACE history (yes vs. no)**	0.86 (0.54–1.37)	0.5434		
**PVI (yes vs. no)**	1.10 (0.73–1.67)	0.6260		
**Child-Pugh (B vs. A)**	1.59 (0.78–3.20)	0.1950		
**BCLC (C vs. B)**	1.06 (0.67–1.67)	0.7845		
**Beyond up-to-7 (yes vs. no)**	1.28 (0.66–2.47)	0.4636		
**Bilirubin (≥17 vs. < 17 μmol/L)**	1.55 (1.02–2.36)	**0.0391**		
**Albumin (≥36 vs. < 36 g/L)**	0.66 (0.42–1.04)	0.0759		
**ALBI (grade 2/3 vs. grade 1)**	1.74 (1.14–2.66)	**0.0091**	1.88 (1.21–2.92)	**0.005**
**AFP (≥400 vs. < 400 ng/mL)**	1.06 (0.67–1.66)	0.8012		

All covariates were time-fixed except for best response, which was time dependent

*AFP* alfa-fetoprotein, *ALBI* albumin-bilirubin, *BCLC* Barcelona Clinic Liver Cancer, *CR* complete response, *ECOG* Eastern Cooperative Oncology Group, *HR* hazard ratio, *PD* progressive disease, *PR* partial response, *PVI* portal vein invasion, *SD* stable disease, *TACE* transarterial chemoembolization

**Fig. 3:**
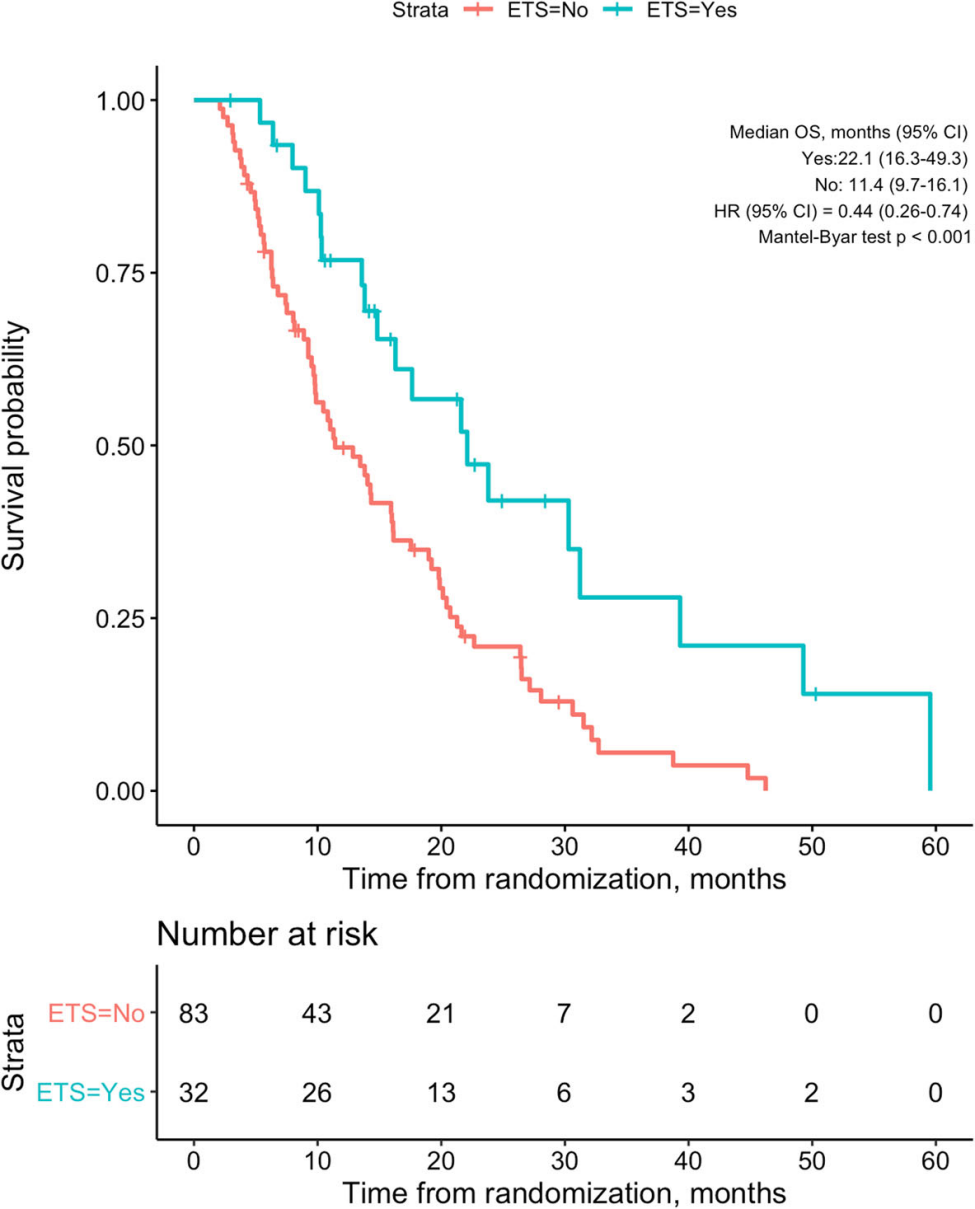
Overall survival of patients with ETS ≥ 20% vs. ETS < 20%. CI, confidence interval; ETS, early tumor shrinkage; HR, hazard ratio; OS, overall survival

Among the 115 patients, ETS ≥20% was achieved in 32 (27.8%) patients. The baseline clinical characteristics of patients with ETS ≥20% compared with ETS < 20% are shown in Table 3. There were no significant differences between patients with ETS ≥20% and ETS < 20%, except for more patients with a history of hepatitis C in the ETS < 20% group (34.9% vs. 15.6%, *p* = 0.041). Median time from randomization to the imaging used for ETS evaluation was 2.2 (range, 0.8–5.2) months. In patients with OR, the median time to ETS was significantly shorter than the time to response (2.2 vs. 3.8 months, *p* = 0.004). Patients with ETS ≥20% had a longer OS (median 22.1 vs. 11.4 months, *p* < 0.001) and PFS (median 8.0 vs. 4.3 months, *p* = 0.034) than patients with ETS < 20% (Fig. 3 and Supplementary Fig. 7). Only 6 (7.2%) of 83 patients with ETS < 20% had an OR during follow-up and an ETS ≥20% was significantly associated with OR (*p* < 0.001). The multivariate analysis with a second model (using ETS instead of OR, Table 4) confirmed ETS (*p* < 0.001) as an independent prognostic factor, besides gender (*p* = 0.004) and ALBI grade (*p* = 0.022).

**Table 3:** Baseline characteristics of patients with ETS ≥20% compared to ETS < 20% (no ETS)

Variable	Total ***n*** = 115	ETS ≥ 20% ***n*** = 32	ETS < 20% ***n*** = 83	***p***-value
**Sex**				
**Female**	15 (13.0%)	7 (21.8%)	8 (9.6%)	0.0808
**Male**	100 (87.0%)	25 (78.1%)	75 (90.4%)	
**Age, years**				
**Mean (SD)**	65.3 (8.7)	67.4 (7.4)	64.5 (9.1)	0.1106
**Median (IQR)**	65.0 (12.0)	67.5 (12.0)	64.0 (13.0)	
**Age category, years**				
≥ **65**	55 (47.8%)	13 (40.6%)	42 (50.6%)	0.3371
**> 65**	60 (52.2%)	19 (59.4%)	41 (49.4%)	
**Ethnicity**				
**Missing**	10 (8.7%)	4 (12.5%)	6 (7.2%)	0.6299
**Hispanic or Latin**	5 (4.8%)	1 (3.1%)	4 (4.8%)	
**Other**	100 (95.2%)	27 (84.3%)	73 (87.9%)	
**Race**				
**Missing**	11 (9.6%)	4 (12.5%)	7 (8.4%)	0.2052
**Black**	2 (1.9%)	0	2 (2.6%)	
**Other**	3 (2.9%)	2 (7.1%)	1 (1.3%)	
**White**	99 (95.2%)	26 (92.9%)	73 (96.1%)	
**ECOG status**				
**0**	90 (78.3%)	24 (75.0%)	66 (79.5%)	0.5986
**1**	25 (21.7%)	8 (25.0%)	17 (20.5%)	
**HCC diagnosis by**				
**Missing**	2 (1.7%)	0	2 (2.4%)	0.6537
**EASL criteria**	50 (43.5%)	12 (37.5%)	38 (45.8%)	
**Histology**	50 (43.5%)	16 (50.0%)	34 (41.0%)	
**Other**	13 (11.3%)	4 (12.5%)	9 (10.8%)	
**Hepatitis B**				
**No**	104 (90.4%)	29 (90.6%)	75 (90.4%)	0.9657
**Yes**	11 (9.6%)	3 (9.4%)	8 (9.6%)	
**Hepatitis C**				
**No**	81 (70.4%)	27 (84.4%)	54 (65.1%)	**0.0419**
**Yes**	34 (29.6%)	5 (15.6%)	29 (34.9%)	
**Alcohol etiology**				
**No**	63 (54.8%)	17 (53.1%)	46 (55.4%)	0.8245
**Yes**	52 (45.2%)	15 (46.9%)	37 (44.6%)	
**Previous therapies**				
**TACE**	28 (24.3%)	6 (18.8%)	22 (26.5%)	0.3851
**TAE**	2 (1.7%)	0	2 (2.4%)	0.3757
**Resection**	21 (18.3%)	8 (25.0%)	13 (15.7%)	0.2454
**RFA**	12 (10.4%)	4 (12.5%)	8 (9.6%)	0.6528
**Brachytherapy**	5 (4.3%)	1 (3.1%)	4 (4.8%)	0.6897
**Max. diameter of largest lesion**				
**Mean (SD)**	68.0 (59.6)	59.1 (41.3)	71.6 (65.3)	0.2127
**Median (IQR)**	57.0 (45.0)	50.0 (38.0)	59.5 (55.0)	
**Portal vein infiltration**				
**Yes**	60 (52.2%)	19 (59.4%)	41 (49.4%)	0.3371
**Baseline metastasis**				
**Yes**	6 (5.2%)	0	6 (7.2%)	0.1182
**BCLC**				
**B**	34 (29.6%)	8 (25.0%)	26 (31.3%)	0.5053
**C**	81 (70.4%)	24 (75.0%)	57 (68.7%)	
**Up-to-7 criterion**				
**Inside**	17 (14.8%)	5 (15.6%)	12 (14.5%)	0.8744
**Outside**	98 (85.2%)	27 (84.4%)	71 (85.5%)	
**Total bilirubin (μmol/L)**				
**Mean (SD)**	16.1 (7.1)	15.3 (7.3)	16.3 (7.1)	0.3959
**Median (IQR)**	14.9 (10.0)	14.0 (9.3)	15.1 (9.9)	
**Albumin g/L**				
**Mean (SD)**	37.8 (8.3)	40.0 (6.1)	37.0 (8.9)	0.2368
**Median (IQR)**	39.0 (7.7)	40.0 (6.0)	38.9 (8.0)	
**ALBI score**				
**Mean (SD)**	−2.5 (0.7)	−2.7 (0.7)	−2.4 (0.8)	0.1644
**Median (IQR)**	−2.5 (0.8)	−2.7 (0.7)	−2.5 (0.8)	
**Child-Pugh score**				
**A**	105 (91.3%)	31 (96.8%)	74 (89.1%)	0.2792
**B**	10 (8.7%)	1 (3.1%)	9 (10.8%)	

*ALBI* albumin-bilirubin, *BCLC* Barcelona Clinic Liver Cancer, *EASL* European Association for the Study of the Liver, *ECOG* Eastern Cooperative Oncology Group, *HCC* hepatocellular carcinoma, *IQR* interquartile range, *RFA* radiofrequency ablation, *SD* standard deviation, *TACE* transarterial chemoembolization, *TAE* transarterial embolization

**Table 4:** Univariable and multivariable analysis of factors associated with overall survival (model with ETS)

Parameter	Univariate analysis		Multivariate analysis	
	HR (95% CI)	***p*** value	HR (95% CI)	***p*** value
**ETS ≥ 20% vs. < 20%**	0.44 (0.26–0.74)	**0.002**	0.44 (0.24–0.69)	**< 0.001**
**Sex (male vs. female)**	0.50 (0.27–0.91)	**0.0241**	0.41 (0.22–0.75)	**0.004**
**Age (≥65 vs. < 65 years)**	1.01 (0.67–1.54)	0.9310		
**ECOG (1 vs. 0)**	0.80 (0.48–1.35)	0.4184		
**Cirrhosis (yes vs. no)**	1.22 (0.63–2.37)	0.5457		
**Hepatitis B etiology (yes vs. no)**	1.22 (0.61–2.44)	0.5677		
**Hepatitis C etiology (yes vs. no)**	1.17 (0.74–1.85)	0.4847		
**Alcohol etiology (yes vs. no)**	0.78 (0.51–1.21)	0.2798		
**TACE history (yes vs. no)**	0.86 (0.54–1.37)	0.5434		
**PVI (yes vs. no)**	1.10 (0.73–1.67)	0.6260		
**Child-Pugh (B vs. A)**	1.59 (0.78–3.20)	0.1950		
**BCLC (C vs. B)**	1.06 (0.67–1.67)	0.7845		
**Beyond up-to-7 (Yes vs. No)**	1.28 (0.66–2.47)	0.4636		
**Bilirubin (≥17 vs. < 17 μmol/L)**	1.55 (1.02–2.36)	**0.0391**		
**Albumin (≥36 vs. < 36 g/L)**	0.66 (0.42–1.04)	0.0759		
**ALBI (grade 2/3 vs. grade 1)**	1.74 (1.14–2.66)	**0.0091**	1.65 (1.08–2.53)	**0.022**
**AFP (≥400 vs. < 400 ng/mL)**	1.06 (0.67–1.66)	0.8012		

All covariates were time-fixed except for ETS, which was time dependent

*AFP* alfa-fetoprotein, *ALBI* albumin-bilirubin, *BCLC* Barcelona Clinic Liver Cancer, *CR* complete response, *ECOG* Eastern Cooperative Oncology Group, *ETS* early tumor shrinkage, *HR* hazard ratio, *PD* progressive disease, *PR* partial response, *PVI* portal vein invasion, *SD* stable disease, *TACE* transarterial chemoembolization

## Discussion

This analysis of data from the SORAMIC trial showed that OR by mRECIST is an independent predictor of OS and PFS in patients receiving sorafenib treatment. The OR rate was 29.5%, and responders had significantly longer median OS than non-responders (30.3 vs. 11.4 months). Besides OR assessment, ETS was an independent predictive factor in our study, identifying responders to treatment earlier than assessment by mRECIST.

Following the demonstration of clinical efficacy in the SHARP trial and Asia-Pacific trial, sorafenib has been the primary treatment of advanced HCC in recent years [2, 3]. Response assessment used RECIST in both these studies, where the OR rate was 2 and 3.3% and failed to capture patients who benefited more from treatment. Failure to differentiate patients who do not benefit from sorafenib may delay a switch in treatment and patients may present with progression that precludes further treatment. In 2010, mRECIST was proposed to address the drawbacks of RECIST caused by the unique complexities of both HCC and its treatment. A meta-analysis confirmed that OR by mRECIST can predict outcome after loco-regional therapies [23]. However, additional data are needed to establish the role of mRECIST in follow-up of systemic therapies [1].

Five retrospective studies used mRECIST for response assessment in patients receiving sorafenib. While four of these studies showed that OR successfully predicts OS [5–8], the other failed to show OS benefit [9]. A combined analysis of two phase II studies showed that OR by mRECIST predicted OS benefit, but, in multivariate analysis, significance was marginally lost [10]. As this was a combined analysis of patients receiving sorafenib and nintedanib, the total number of patients receiving sorafenib was only 63. Another limitation of these studies was the utilization of a simple comparison of responders with non-responders, leading to guarantee-time bias or immortal time bias. Recently, subgroup analysis of a phase III study conducted in Japan showed superior OS (27.2 vs. 8.9 months, *p* < 0.001) in patients with OR after sorafenib treatment [12]. In this analysis, landmark analyses, Mantel-Byar test were used to eliminate guarantee-time bias, and OR was incorporated into multivariable analysis as a time-dependent variable, as in our study. Our study confirms these findings in a Western population. Univariate analysis revealed gender, total bilirubin, and ALBI grade as predictive factors, besides OR, and multivariate analysis confirmed these findings (bilirubin was excluded because of interactions with ALBI). Patients with ALBI grade 2 and 3 had worse OS than those with grade 1 in our study (HR, 1.88 [95% CI, 1.21–2.92], *p* = 0.005), and this finding is in agreement with previous reports [24, 25]. A surprising result was the better OS in male patients in our study, unlike most previous reports [6, 12]. However, this may be the result of the small number of female patients (15, 13%).

ETS has been shown to predict treatment outcome in various tumor types, but, to date, only one study has evaluated ETS in patients with HCC. Takahashi et al. showed that patients with ETS ≥10% had better OS and PFS than patients with ETS < 10% after lenvatinib treatment [19]. In that study, ETS was defined based on RECIST criteria, and a cut-off of 10% was chosen. However, in our study, measurement of enhancing tumor instead of overall diameter was used to overcome problems related to the unique features of HCC described above, and a cut-off of 20% was chosen, as most previous authors have done [16–18]. ETS can detect patients who do not benefit from treatment earlier than response assessment with mRECIST, and makes possible necessary therapeutic adjustments before progression is encountered. Early detection of patients who do not benefit from sorafenib treatment gains greater importance in the light of other emerging, effective second-line drugs [13, 15]. With early sequencing of treatment, progression precluding further treatment or associated with shorter post-progression survival (i.e. new macrovascular invasion) can be avoided.

This study has some limitations. First, follow-up imaging was not conducted according to a standardized protocol, since follow-up imaging was at the discretion of the local investigator. However, baseline imaging of all patients was done in a standardized fashion within the diagnostic arm of the SORAMIC trial, and the images used for follow-up assessment were high-quality state-of-the-art triphasic images. Second, follow-up imaging was not available in 30.9% of the patients who received sorafenib treatment in the SORAMIC trial. There is an inherent risk of selection of responders and patients with longer survival. Patients with poorer performance status or liver function might not have undergone cross-sectional follow-up imaging due to earlier deterioration in their clinical situation. The OS of the patients included in this study was a little longer than the OS of the sorafenib arm in the SORAMIC study (14.3 vs. 11.4 months). Nevertheless, this study was conducted on patients using prospectively collected data from the phase II SORAMIC trial, and it showed, for the first time in a Western population, a correlation between OR by mRECIST and OS in HCC patients receiving sorafenib, and also, for the first time, a correlation between ETS and OS in the same cohort.

## Conclusion

OR assessments by mRECIST and ETS in HCC patients receiving sorafenib monotherapy are associated with treatment outcome and survival. Both assessments can be used to identify patients who do not benefit from sorafenib and to guide the decision-making process in the era of effective second-line therapies. However, the use of ETS needs further validation.

## Supplementary Information

**Additional file 1: Supplementary Fig. 1.** Consort diagram.

**Additional file 2: Supplementary Fig. 2.** Examples for image analysis.

**Additional file 3: Supplementary Fig. 3.** Overall survival of patients with partial response according to depth of response (DpR) more or less than the median DpR. CI, confidence interval; HR, hazard ratio.

**Additional file 4: Supplementary Fig. 4.** Progression-free survival of patients with ETS ≥ 20% vs. ETS < 20%. CI, confidence interval; ETS, early tumor shrinkage; HR, hazard ratio; PFS, progression-free survival.

**Additional file 5: Supplementary Fig. 5.** Overall survival of patients according to best response. BR, best response; CI, confidence interval; CR, complete response; HR, hazard ratio; PR, partial response; PD, progressive disease; SD, stable disease.

**Additional file 6: Supplementary Fig. 6.** Landmark Kaplan-Meier curve as function of tumor response at 6 months. OR, objective response.

**Additional file 7: Supplementary Fig. 7.** Landmark Kaplan-Meier curve as function of tumor response at 12 months. OR, objective response.

## Data Availability

Data are available through corresponding author upon reasonable request.

## Abbreviations

ALBI: Albumin-Bilirubin
BCLC: Barcelona Clinic Liver Cancer
CI: Confidence interval
CR: Complete response
CT: Computed tomography
DpR: Depth of response
ETS: Early tumor shrinkage
HCC: Hepatocellular carcinoma
HR: Hazard ratio
mRECIST: modified Response Evaluation Criteria in Solid Tumors
MRI: Magnetic resonance imaging
OR: Objective response
OS: Overall survival
PD: Progressive disease
PFS: Progression-free survival
PR: Partial response
SD: Stable disease
