# Supplementary figures and images for: Early tumor shrinkage and response assessment according to mRECIST predict overall survival in hepatocellular carcinoma patients under sorafenib
Source: Cancer Imaging. 2022 Jan 4;22:1. doi: 10.1186/s40644-021-00439-x (PMC8725442; doi:10.1186/s40644-021-00439-x)

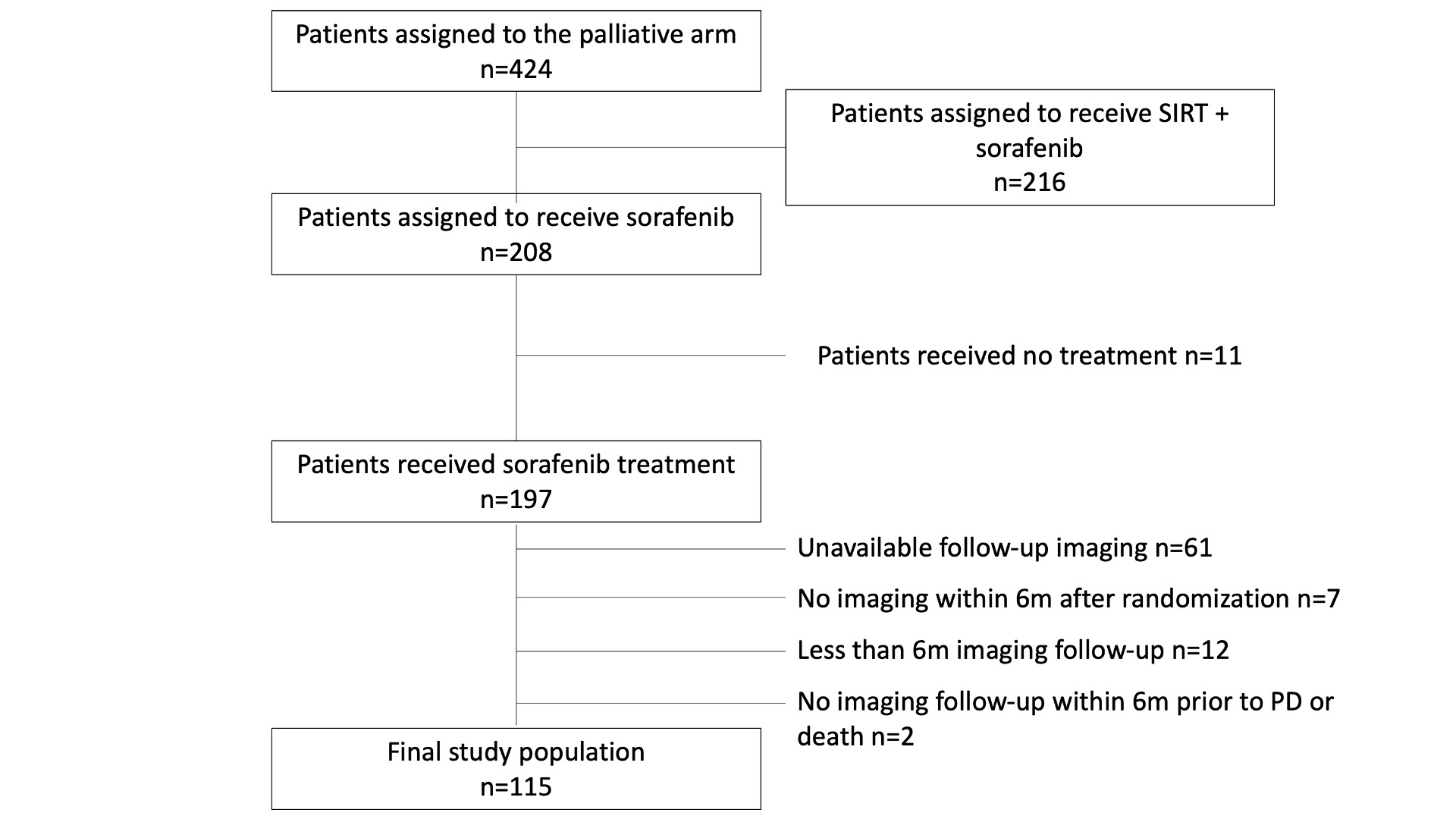

Supplement: Supplementary file 1 — Additional file 1: Supplementary Fig. 1. Consort diagram. [file 40644_2021_439_MOESM1_ESM.jpg]

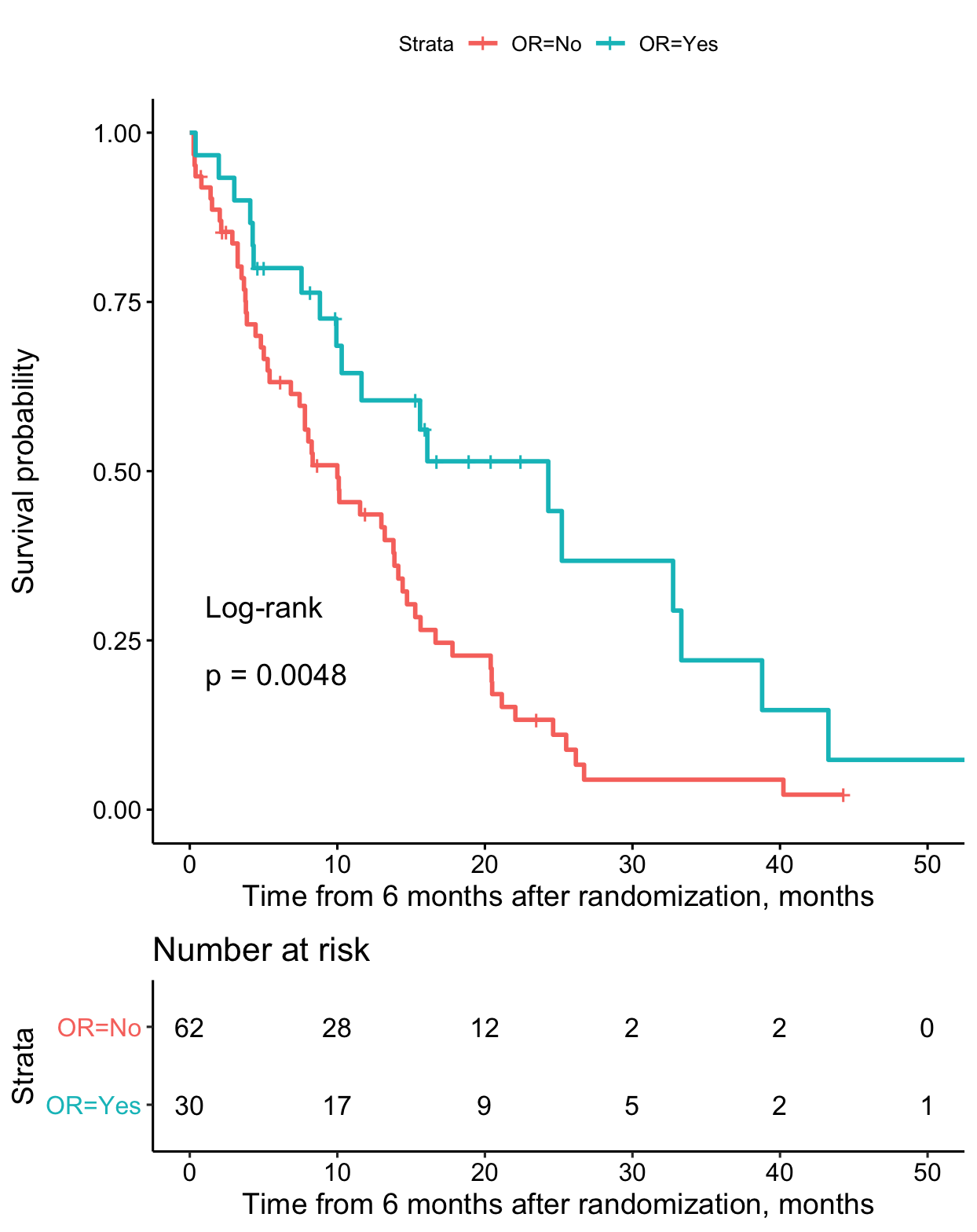

Supplement: Supplementary file 3 — Additional file 3: Supplementary Fig. 3. Overall survival of patients with partial response according to depth of response (DpR) more or less than the median DpR. CI, confidence interval; HR, hazard ratio. [file 40644_2021_439_MOESM3_ESM.png]

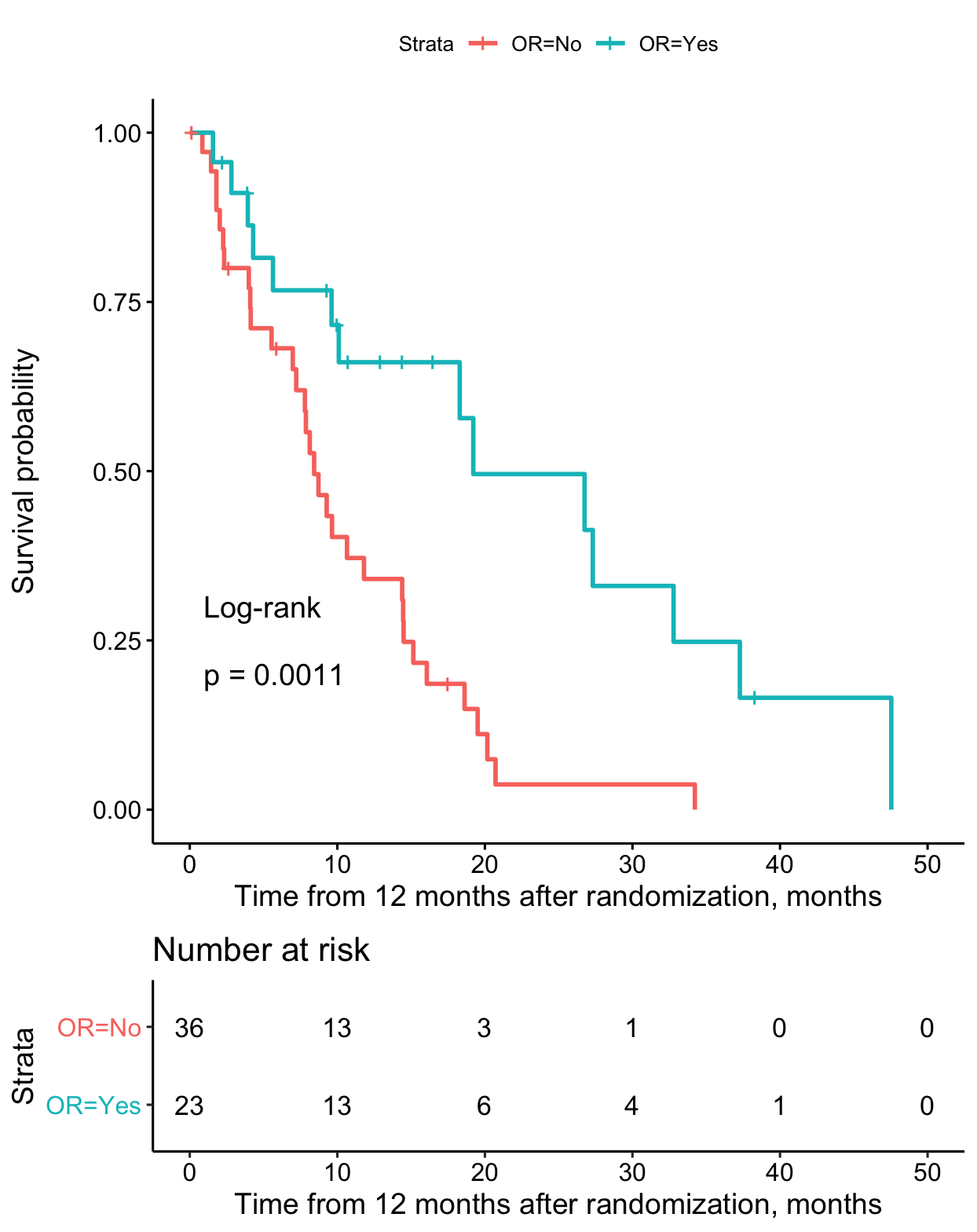

Supplement: Supplementary file 4 — Additional file 4: Supplementary Fig. 4. Progression-free survival of patients with ETS ≥ 20% vs. ETS < 20%. CI, confidence interval; ETS, early tumor shrinkage; HR, hazard ratio; PFS, progression-free survival. [file 40644_2021_439_MOESM4_ESM.png]

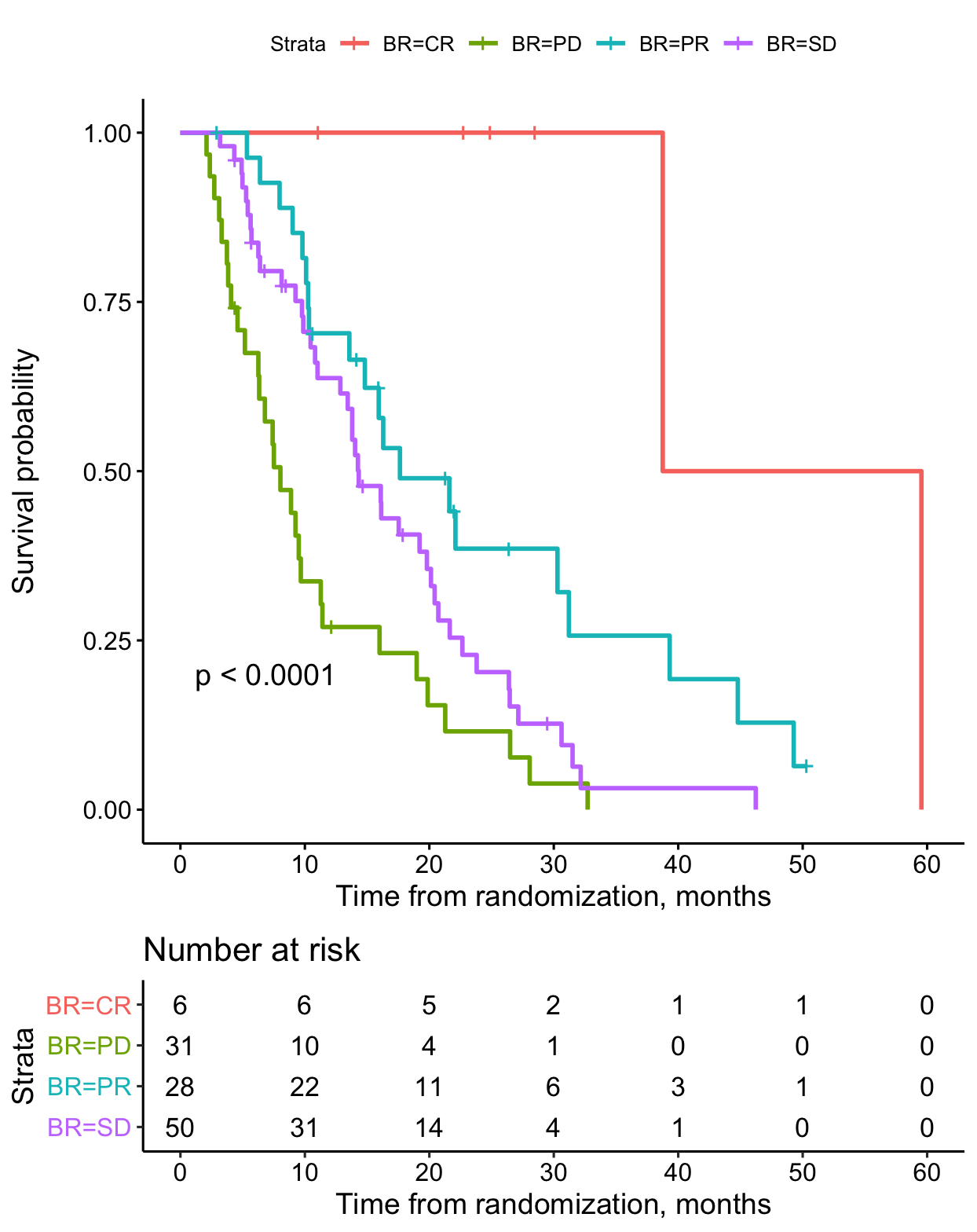

Supplement: Supplementary file 5 — Additional file 5: Supplementary Fig. 5. Overall survival of patients according to best response. BR, best response; CI, confidence interval; CR, complete response; HR, hazard ratio; PR, partial response; PD, progressive disease; SD, stable disease. [file 40644_2021_439_MOESM5_ESM.png]

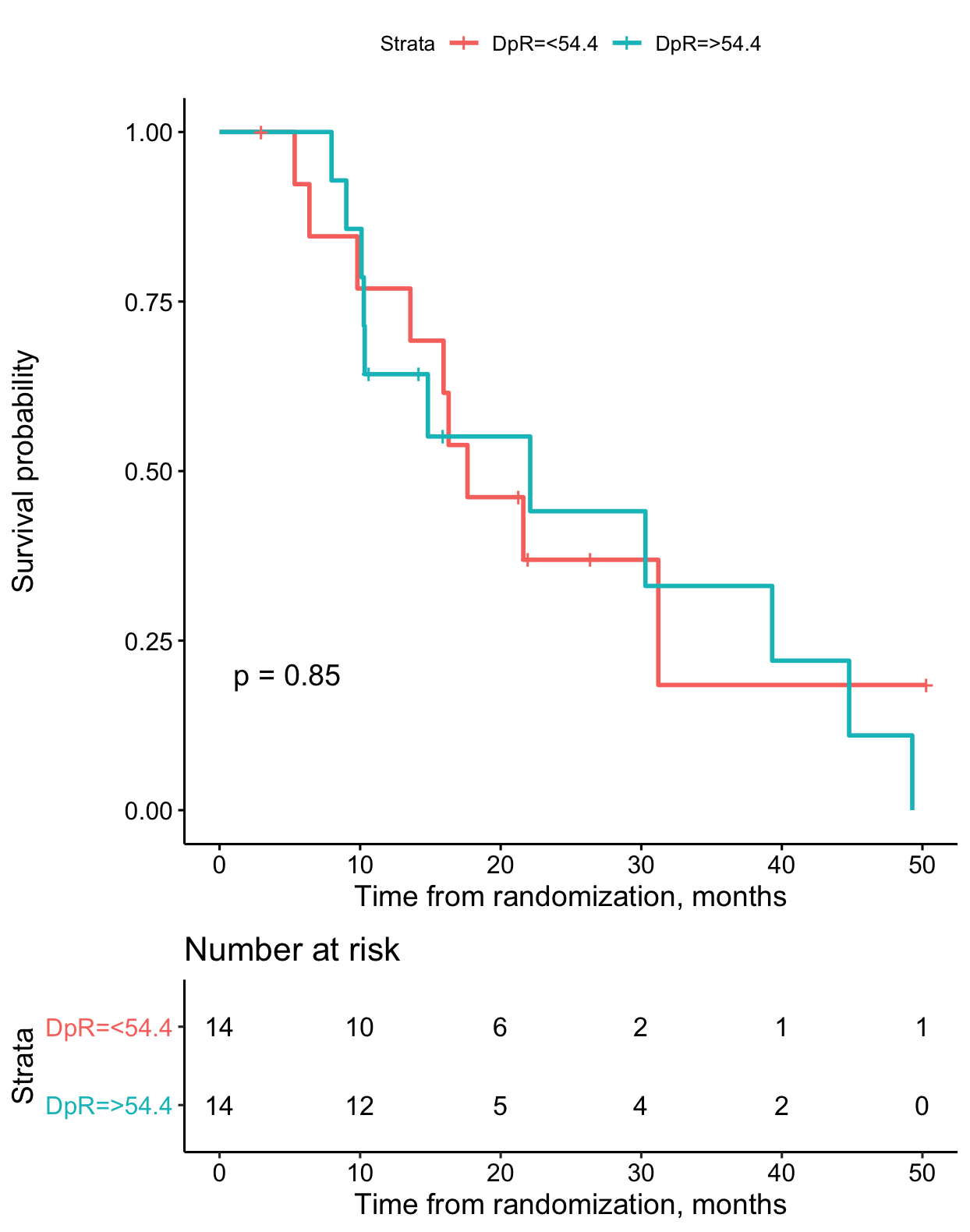

Supplement: Supplementary file 6 — Additional file 6: Supplementary Fig. 6. Landmark Kaplan-Meier curve as function of tumor response at 6 months. OR, objective response. [file 40644_2021_439_MOESM6_ESM.png]

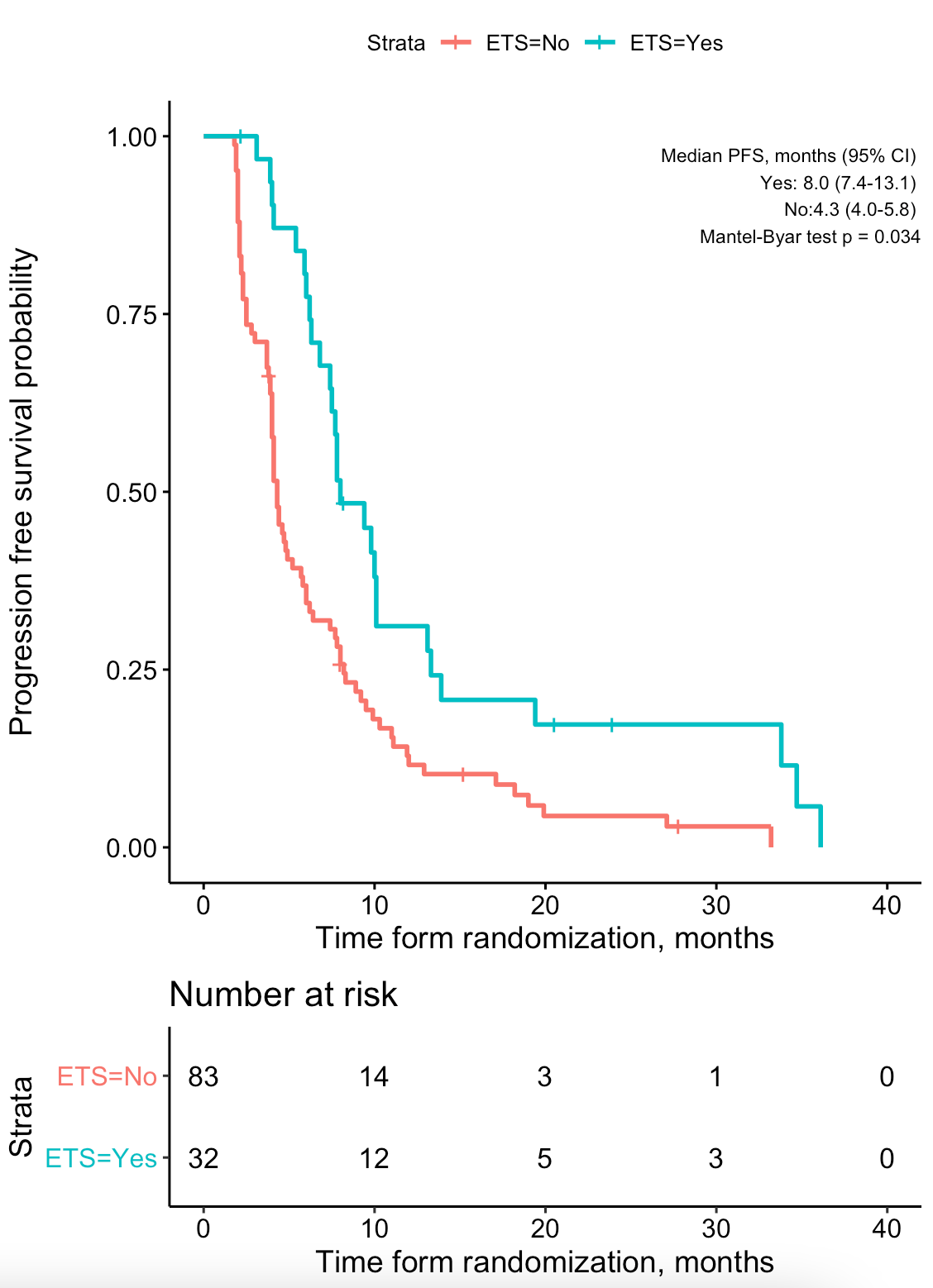

Supplement: Supplementary file 7 — Additional file 7: Supplementary Fig. 7. Landmark Kaplan-Meier curve as function of tumor response at 12 months. OR, objective response. [file 40644_2021_439_MOESM7_ESM.png]
