# Supplementary material for: Early tumor shrinkage and response assessment according to mRECIST predict overall survival in hepatocellular carcinoma patients under sorafenib
Source: Cancer Imaging. 2022 Jan 4;22:1. doi: 10.1186/s40644-021-00439-x (PMC8725442; doi:10.1186/s40644-021-00439-x)
Supplement: Supplementary file 2 — Additional file 2: Supplementary Fig. 2. Examples for image analysis. [file 40644_2021_439_MOESM2_ESM.pdf]

Supplementary figure 1a.

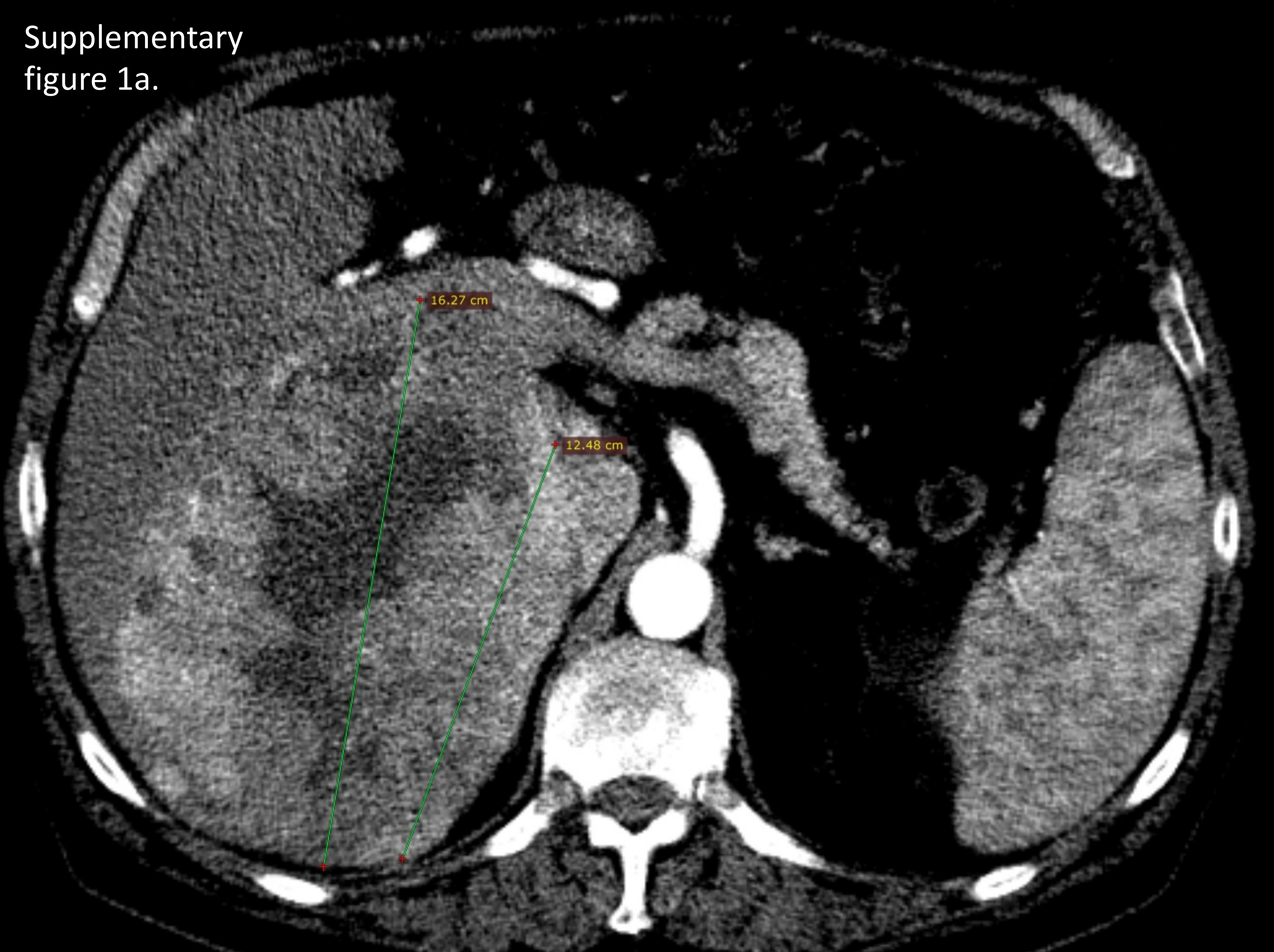

Baseline arterial phase CT image shows a 16.2 cm lesion with centrally non-enhancing necrosis. The measurement of the longest viable tumor diameter according to mRECIST is 12.5 cm.

Supplementary figure 1b.

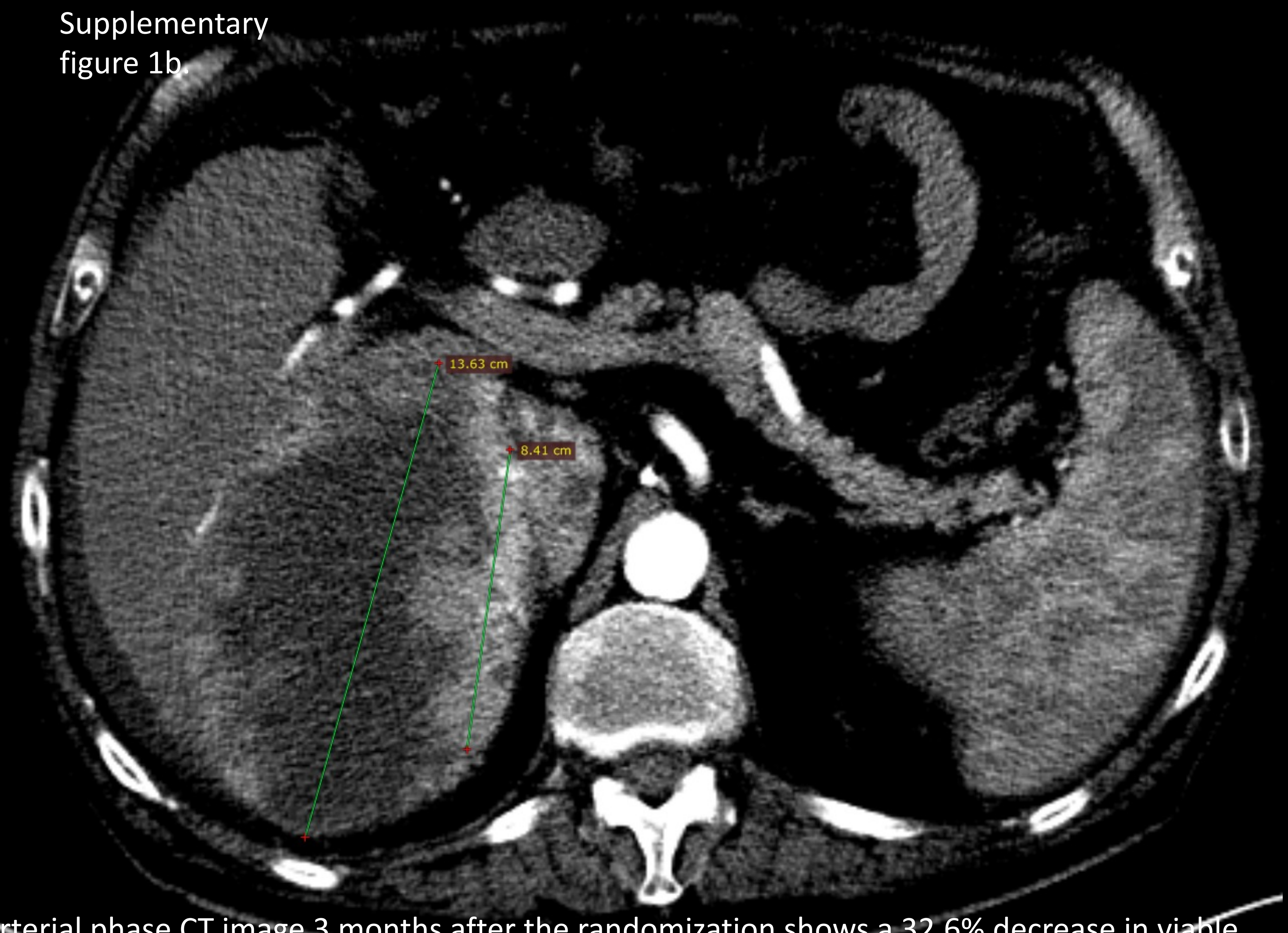

Arterial phase CT image 3 months after the randomization shows a 32.6% decrease in viable tumor diameter according to mRECIST (partial response).

Supplementary
figure 2a.

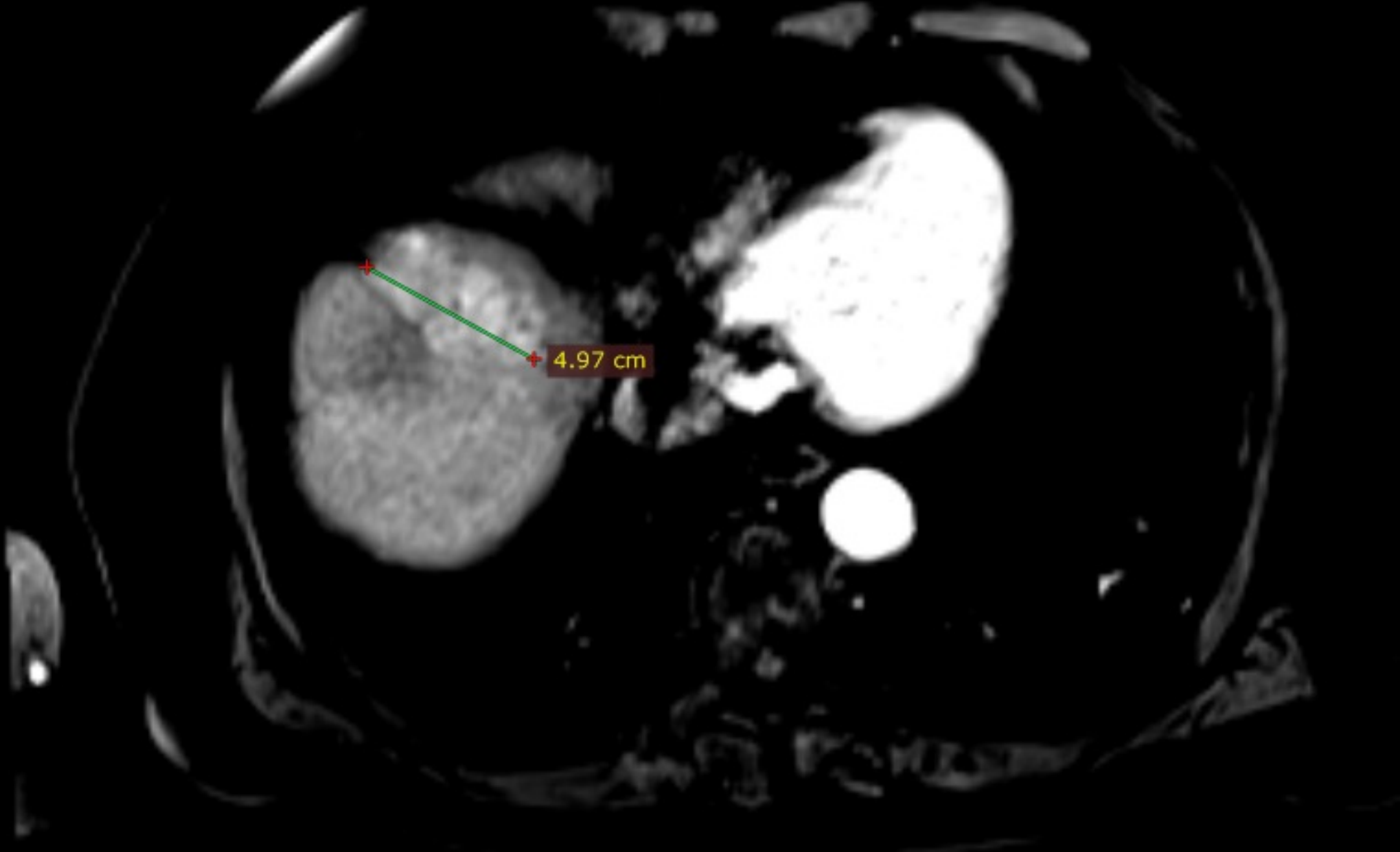

Baseline arterial phase MR image shows a lesion with a diameter of 5 cm next to the previous ablation zone.

Supplementary figure 2b.

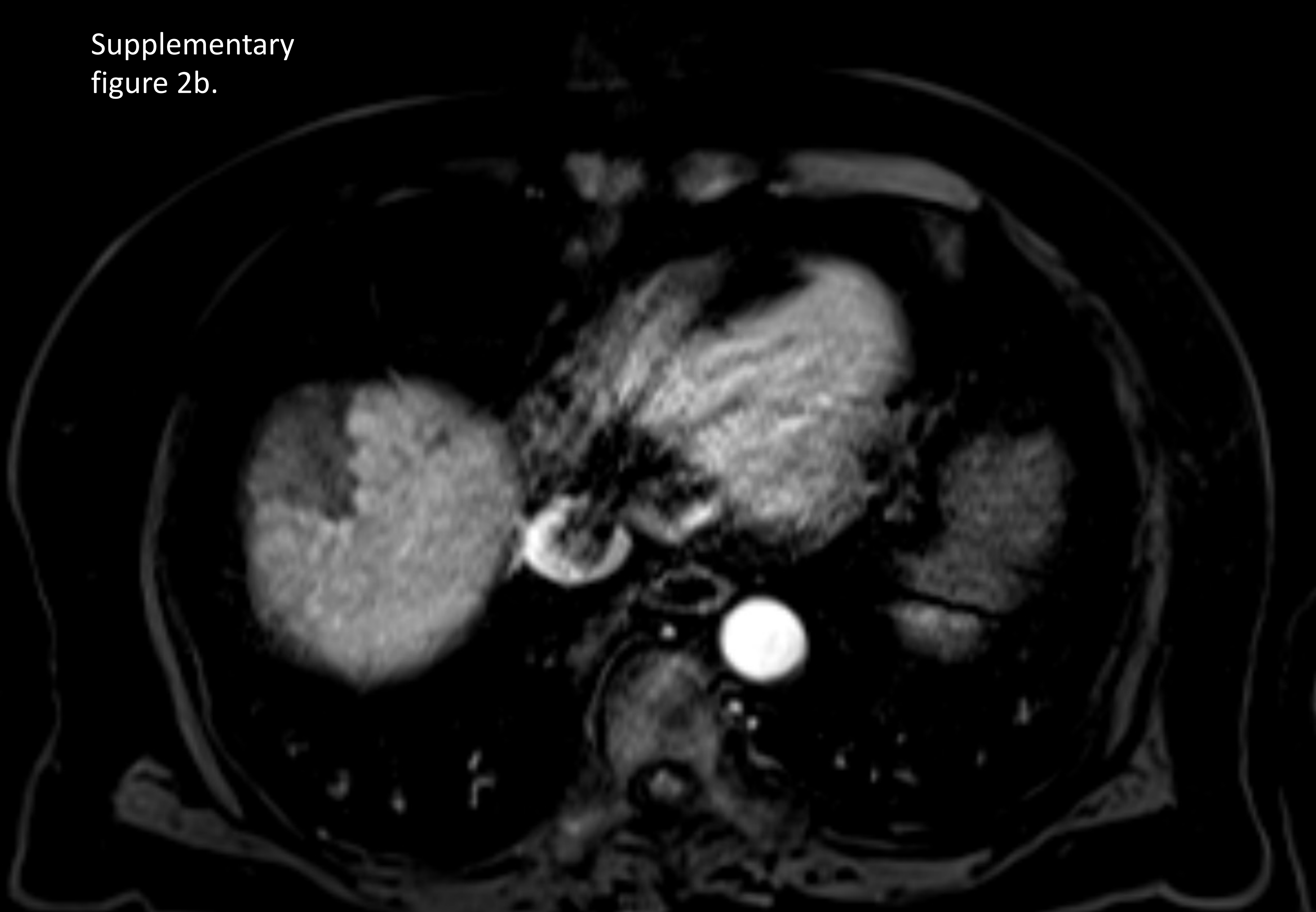

Follow-up arterial phase MR image at 8 months shows complete devascularization of the lesion (complete response) and other lesions in the liver (not shown).

Supplementary figure 2c.

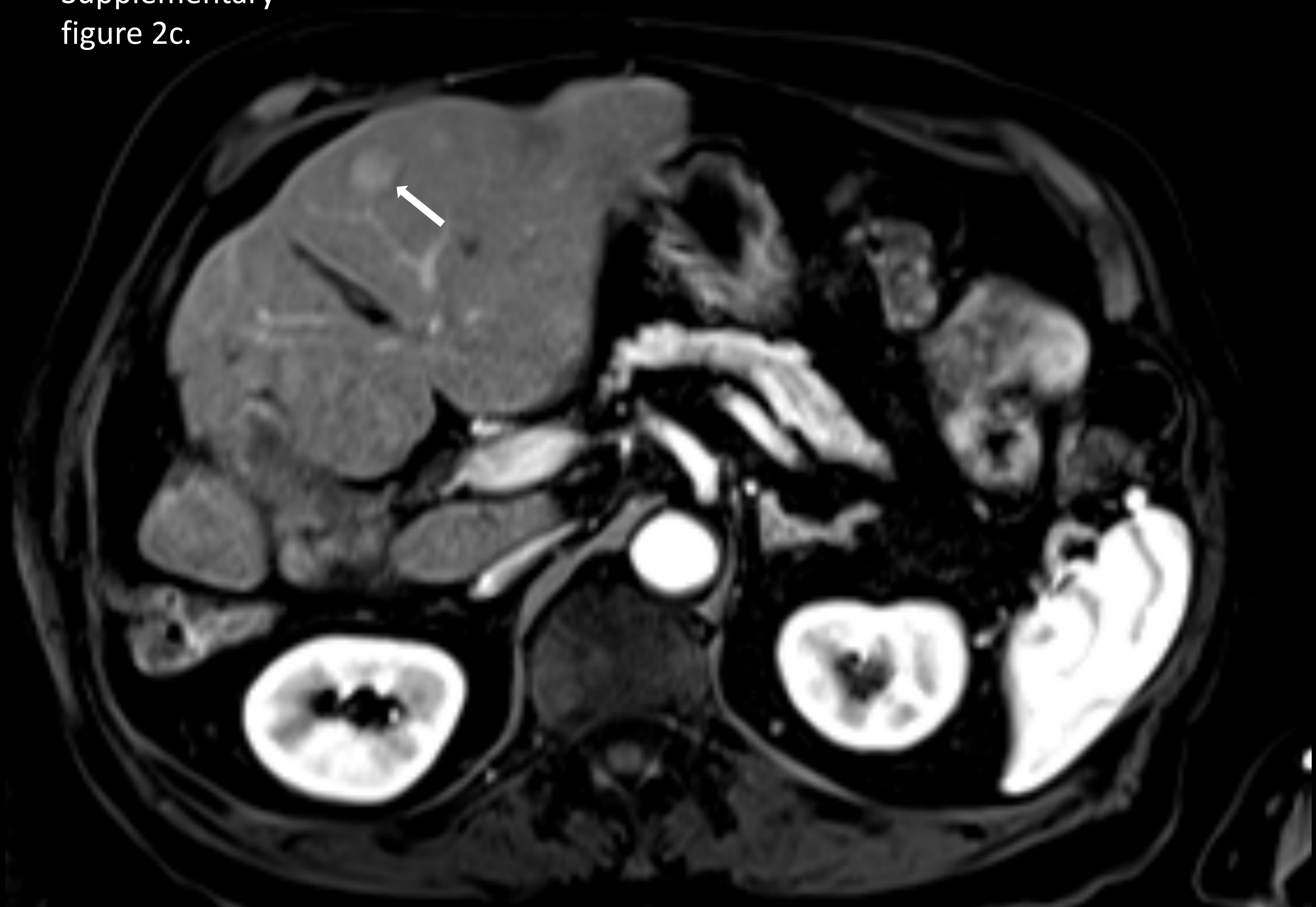

Follow-up arterial phase MR image 13 months after the randomization shows new arterial phase enhancing lesions (arrow; progressive disease).
